# Fecal Microbiota Transplantation Attenuates Frailty *via* Gut-Muscle Axis in Old Mice

**DOI:** 10.14336/AD.2024.0321

**Published:** 2024-03-21

**Authors:** Mengpei Zhu, Yumei Huang, Ziwen Wang, Ze Jin, Jiali Cao, Qiangqiang Zhong, Zhifan Xiong

**Affiliations:** ^1^Institute of Geriatric Medicine, Liyuan Hospital, Tongji Medical College, Huazhong University of Science and Technology, Wuhan, China.; ^2^Division of Gastroenterology, Liyuan Hospital, Tongji Medical College, Huazhong University of Science and Technology, Wuhan, China.

**Keywords:** Frailty, FMT, Gut-muscle axis, SCFAs, Gut microbiota

## Abstract

Targeting adverse pathogenic gut microbiota regulation through fecal microbiota transplantation (FMT) may restore health and has been validated in some aging-related diseases. However, the mechanisms of the gut microbiota's role in frailty and whether modulation of the gut microbiota can treat age-related frailty remain largely unknown. To assess the effects of FMT on frailty, we used bidirectional fecal microbiota transplantation in young and old mice. We demonstrated that fecal bacteria transplanted from old mice into young mice reduced body weight and grip strength (p=0.002), and led to elevated inflammatory factors in young mice, but had no significant effect on intestinal barrier function. Notably, FMT treatment in older mice not only improved frailty (grip strength: p=0.036, low physical activity: p=0.020, running speed: p=0.048, running time: p=0.058, frailty score: p=0.027) and muscle mass, but also improved intestinal ecological imbalances, intestinal barrier function, and systemic inflammation (serum TNF-α: p=0.002, and IL-6: p<0.001). KEGG enrichment analysis of fecal metabolites showed that FMT may ameliorate frailty through the sphingolipid metabolism pathway. In addition, aged mice given FMT treatment showed a significant increase in the abundance of SCFA-producing bacteria and increased levels of short-chain fatty acids (butyric acid: p=0.084, propionic acid: p=0.028). Subsequent further verification found that FMT ameliorating frailty may be achieved through SCFAs metabolism. Another mechanism study found that FMT reduces lipopolysaccharide levels (p<0.001), thereby inhibiting the TLR4/NF-κB signaling pathway and its downstream pro-inflammatory products. Therefore, regulating SCFAs metabolism by altering gut microbial composition and targeting the gut-muscle axis with LPS/TLR4 pathways may be potential strategies to treat frailty in older adults.

## INTRODUCTION

Undoubtedly, frailty is an emerging global health burden as aging becomes more prominent [1]. Frailty is a complex age-related clinical syndrome characterized by a decline in function and reserve of multiple physiological systems [2] which can damage the physical function of older adults and significantly increase their risk of disabilities and other poor outcomes. Early interventions for frailty can be effective in reducing adverse outcomes and reducing the cost of healthcare and the social burden on families as the world enters an aging society. Effective interventions for frailty are currently lacking and are focused on exercise, nutrition, and integrated multidisciplinary interventions, but their efficacy is highly variable. Therefore, there is an urgent need to further identify new therapeutic targets to enhance interventions for frailty.

Interactions between gut microbes and humans occur at every stage of life [3]. The human gut microbiota can influence host physiology by regulating a variety of processes, including nutrient absorption, inflammation, oxidative stress, immune function, and anabolic homeostasis. With age, the microbiome shifts to a pro-inflammatory phenotype, while age-related intestinal mucosal barrier dysfunction may lead to the translocation of microorganisms and their products into the systemic circulation, resulting in a systemic inflammatory response [4]. In addition, studies have found a reduction in host-beneficial microbiota in older populations, such as beneficial bacteria that maintain mucus production and mucosal barrier integrity [5-7]. Notably, altered gut microbiota composition in older people is associated with increased chronic low-grade inflammation and susceptibility to age-related chronic diseases [8]. The results of several studies with large populations suggested that frailty, as a syndrome of aging, is associated with a reduction in the biodiversity of the fecal microbiota. Many pathological changes in microbial taxonomic abundance have been reported in frailty patients. A meta-analysis of 10 case-control studies and 1 cohort study showed that the microbiota of frail older people is characterized by a reduction in phylum Firmicutes, with Dialister, Lactobacillus, and Ruminococcus being the prominent genera [9]. On the other hand, healthy controls exhibited *Eubacterium* at the general level. It was also found that frail older people had lower gut microbiota diversity and a lower abundance of short-chain fatty acids (SCFAs) producers [9]. In terms of intestinal permeability, frail intestinal barrier function is impaired, which can lead to intestinal leakage, pro-inflammatory microbial products such as lipopolysaccharides (LPS), and cytokines crossing the damaged barrier into circulation, resulting in systemic inflammation [10]. Overall, gut microbiota dysbiosis plays a key role in the development of frailty. The gut microbiota regulates LPS production, SCFAs metabolism, and various metabolites affecting host organism metabolism, including skeletal muscle tissue, and may play a role in the etiology of sarcopenia [11].

The physical manifestations of frailty are often characterized by sarcopenia, and muscle health plays a key role in the vicious cycle of frailty [12]. Although the causal relationship remains uncertain, results from animal and human studies support the presence of a gut-muscle axis actively involved in the pathophysiology of frailty and sarcopenia, i.e. the gut-muscle axis [13]. Emerging research suggested that fecal transplants can regulate the gut microbiota and have therapeutic benefits in preventing inflammation-related tissue decline in later life [8]. A growing body of data suggested that targeting the regulation of adverse pathogenic gut microbiota through fecal microbiota transplantation (FMT) may restore health under a range of harmful inflammatory conditions [4]. Xiao et al. found that FMT can improve chronic cerebral hypoperfusion by increasing SCFA levels [14]. In addition, Parker et. al found that FMT between young and aged mice reverses hallmarks of the aging gut, eye, and brain in older mice [8]. Kim et al. revealed that the young-derived gut microbiota rejuvenates the physical fitness of the aged by altering the microbial composition of the gut and gene expression in muscle and skin [15]. Zhang et al. found that young microbiota colonized older rats inhibited the development of age-related atrial fibrillation [16]. Coincidentally, Zeng et al. found that FMT from young mice rejuvenated senescent hematopoietic stem cells by suppressing inflammation [17]. It has also been found that FMT can improve cognitive impairment and Parkinson's syndrome in older mice through the gut-brain axis [18, 19]. However, the mechanisms of the gut microbiota's role in frailty and whether modulation of the gut microbiota can treat age-related frailty remain largely unknown.

In this study, we used bidirectional fecal transplantations in young and old mice to assess the effect of the gut microbiota and its associated metabolites on frailty in old mice, elucidate its underlying mechanisms, and further explore the potential role of the microbiota-gut-muscle axis in the pathogenesis of frailty.

## MATERIALS AND METHODS

### Animals

Adult (5-6 months) and aged (22-23 months) male C57BL/6J mice were housed under specific pathogen-free conditions as described in the Supplemental Methods. There were 6 mice in each group. All animal experiments were conducted under guidelines and approved by the Experimental Animal Ethics Committee of Huazhong University of Science and Technology (IACUC Number 3650).

### FMT

Before FMT, the existing microbiota was depleted by a 3-day broad-spectrum antibiotic cocktail regimen as described in the Supplemental Methods [8]. Post-antibiotic washouts, recipients were rehoused in heterochronic donor cages containing soiled bedding and fecal pellets and were orally gavaged with 200 μL microbiota suspension, every other day for 6 weeks [8]. Fecal slurries were prepared from donor mice by pooling fecal pellet material from two mice per cage (chosen randomly within each cage). At pellet collection, young or aged donor mice were placed in clean cages alone and fecal particles were collected within 10 minutes. Fresh fecal pellets (300 mg) were suspended in 2 mL phosphate-buffered saline. The solution was vigorously mixed for 10s using a bench-top vortexer, followed by centrifugation at 800 g for 5 minutes and collection of the supernatant. Donor stool was freshly prepared within 1 h before gavage administration on the day of transplantation to prevent changes in bacterial composition.

### SCFAs supplementation

Aged mice were continuously given SCFAs (a mixture of 67.5 mM sodium acetate, 25.9 mM sodium propionate, and 40 mM sodium butyrate), or a salt-matched control solution (133.4 mM sodium chloride) in drinking water ad libitum continuously for 6 weeks.

### Physical frailty phenotype

The physical frailty phenotype criteria were assessed at the end of the experimental intervention and included the evaluation of 5 abilities: loss of weight, weakness (grip strength: lowest 20%), slowness (running speed: slowest 20%), poor endurance (running time/distance to exhaustion: lowest 20%) and low physical activity level (the time to fall in Rota Rod: lowest 20%) as previously described [20, 21]. We assessed frailty using a score based on the clinical phenotype of frailty, and mice with three or more criteria were categorized as frality [20].

### Body Weight

Animal body weight was recorded using an electronic scale.

### Grip Strength Test

The Grip Strength Meter (Shanghai Xinxin Information Technology Co., LTD, XR-6C) was used to measure the maximum force displayed by a mouse. The mice were placed on the grip strength meter and after making sure that the mice grasped the metal net with their forelimbs and then they were pulled backward in the horizontal plane with a balanced force until the mice released the grip plate. Each mouse was tested three times, and the peak grip strength and grip strength curve were recorded.

### Motor coordination

Motor coordination was assessed with a Rota Rod test (Shanghai Xinxin Information Technology Co., LTD, XR-6C). Set the Rota-Rod for an initial speed of 0 rpm, uniform acceleration for 5 minutes, and a final speed of 40 rpm and test it after 3 days of acclimatization training. Set the speed of the rotarod at 40 rpm and record the time for each mouse to fall off the rod. Each mouse was tested three times.

### Incremental Treadmill Test

The animals were submitted to a graded intensity treadmill test (Shanghai Xinxin Information Technology Co., LTD, XR-PT-10B) to determine their endurance (running time) and running speed. Mice were acclimatized to running for 3-4 days, 1-3 times a day, at 5-10 minute intervals. During the test, after a warm-up period, the treadmill band velocity was increased until the animals were unable to run further. The initial bout of 6 minutes at 6 m/min was followed by consecutive 2 m/min increments every 2 minutes. Encourage the continuation of the test by gently tapping the back of any mouse that starts to fall behind on the treadmill using a cotton swab. Recorded the running time (endurance) and the maximal running speed after the third consecutive time the mouse needs extra encouragement.

### Novel Object Recognition test

The novel object recognition test was designed to assess the cognitive ability of mice. During the first phase, the mice were allowed to orient themselves in the environment for 30 min before exploring a nontransparent plastic box for 5-10 min. In the test phase, two identical objects were placed in opposite positions in the box, and the mice were allowed to explore them for 10 min. After a one-hour interval, the mice were returned to the box, and one of the familiar objects was replaced with a new novel object. This phase lasted for 10 min and the box, and objects were cleaned with 75% ethanol after each session. Only when the mice were touching the object with their nose and/or directing their nose to the object within 2 cm, was the exploration time of each object recorded. The preference scores were calculated as the time spent exploring new objects divided by the total time spent exploring familiar and new objects.

### Fecal DNA extraction and 16S RNA sequencing

6-8 fresh fecal pellets were collected from each mouse and immediately stored in a - 80°C refrigerator for further analysis. Microbial genomic DNA was extracted from fecal samples by the Fecal Rapid DNA Kit according to the manufacturer's instructions. After the extraction was completed, the DNA concentration was detected by Qubit, and the integrity of the extracted genomic DNA was detected by 1% agarose gel electrophoresis. The V3-V4 regions of the microbial 16S RNA were amplified with the paired primers (forward primer: 5′-CCTACGGGN GGCWGCAG-3′; reverse primer: 5′-GGACTACH VGGGTWTCTAAT-3′). The following condition was used: 95 °C for 3 min, followed by 25 cycles at 95 °C for 30 s, then 55 °C for 30 s, 72 °C for 15 s, and a final extension at 72 °C for 5 min. All the quantified amplicons were pooled together at equalized concentrations for Illumina MiSeq sequencing (Illumina, Inc., CA, USA). Experiments including DNA extraction, PCR amplification, quality assessment, amplicon library construction, and high-throughput sequencing were performed by Wuhan Genecreate Biological Engineering CO., LTD.

### Hematoxylin and eosin staining and AB-PAS

The colons and gastrocnemius muscles are dehydrated and embedded in paraffin. Sections (4 μm thick) were stained with hematoxylin and eosin (H&E) and AB-PAS. To quantify myofibre size, the visual fields of 4-5 representative fibers were randomly selected for each sample to outline the muscle fibers, and their cross-sectional area was determined using ImageJ-ProPlus software. The histological score was blindly assessed by 2 researchers. Colonic H&E staining was scored using the following four parameters: (1) atrophy of the crypt; (2) polymorphonuclear leukocyte and monocyte infiltration in the crypt; (3) number of GCs and mucus production in the crypt; and (4) Lymphocyte infiltration at the base of the crypt and mucosal base. Scoring criteria: normal = 0 points, mildly increased = 1 point, moderately increased = 2 points, significantly increased = 3 points.

### Transmission electron microscopy (TEM)

Each colon sample was cut into cubes of 1 mm^3^ in size and placed into fixative at 4 °C for 4 hours. The tissues were then fixed then dehydrated with gradient alcohol as described in the Supplemental Methods. Subsequently, the tissues were embedded in resin and baked at 60°C for 48 h to cure. A transmission electron microscope (Hitachi TEM system) was utilized to observe the ultrastructure of tight junctions in the colon.

### RT-qPCR

Total RNA was isolated with an RNA-easy Isolation Reagent (Vazyme, Nanjing). Reverse transcription was operated with HiScript II Reverse Transcriptase (Vazyme, Nanjing). The relative mRNA expression of all genes was determined by the 2- ΔΔCt method. Data shown are relative expression levels of mRNA compared with the Y group. Primer sequences used in this study are shown in Table 1.

**Table 1:** Primer sequences used for RT-qPCR.

Target genes	Forward primer	Reverse primer
**TNF-α** **IL-1β** **IL-6** **TLR4** **NF-κB** **GAPDH**	AAGACACCATGAGCACAGAAAGC CACCTCACAAGCAGAGCACAAG ACCAGAGGAAATTTTCAATAGGC CTGTGGACAAGGTCAGCAACTC TATTAGAGCAACCAAAACAG TCAACGGCACAGTCAAGG	GCCACAAGCAGGAATGAGAAGAG GCATTAGAAACAGTCCAGCCCATAC TGATGCACTTGCAGAAAACA TCTGTTGGGAGTGGTATCCTCTGTG ATACCCCGTCCTCACAGT TTAGTGGGGTCTCGCTCC

Notes: TNF-α: Tumor necrosis factor α, IL-1β: Interleukin-1β, IL-6: Interleukin-6, TLR4: Toll-like receptor 4, NF-κB: nuclear factor-kappaB.

### Western blot

For total protein extraction, RIPA lysis buffer (G2002, service, China) with protease inhibitor cocktail (G2006, service, China) and phosphatase inhibitors mixture (P1260, service, China) was utilized to lyse the tissues. Then, the lysate was centrifuged at 12,000 g, 4°C for 15 min to obtain the total protein. The protein concentrations were detected by the BCA Protein Assay Kit (biosharp, China), and a western blot was performed using the sodium dodecyl sulfate-polyacrylamide gel electrophoresis (SDS-PAGE) method. The transferred membranes were incubated at 4°C overnight with the following primary antibodies: mouse anti-TLR4 antibody (1:1000, 66350-1, ProteinTech)), rabbit anti-NF-κB p65 antibody (1:1000, D14E12, CST), rabbit anti-p-NF-κB p65 antibody (1:1000, 8242 CST, USA), rabbit anti-claudin-1 antibody (1:1000, 28647-1-AP, ProteinTech), rabbit anti-occludin antibody (1:5000, 27260-1-AP, ProteinTech). The membranes were then incubated with the corresponding secondary antibodies, including HRP anti-mouse antibody (1:10000, ProteinTech), and HRP anti-rabbit antibody (1:10000, ProteinTech) for 1 h at room temperature. The blots were visualized using the Tanon 5200 chemiluminescence system, and the densities were analyzed by Image J software.

### Enzyme-linked immunosorbent assay

The enzyme-linked immunosorbent assay (ELISA) kits used to detect the levels of mouse LPS, TNF-α, and, IL-6 were purchased from Bioswamp, Wuhan, China. The experimental procedures were carried out according to the manufacturer’s instructions. Measurements were repeated three times for each sample.

### Determination of SCFAs levels

200 mg of fecal samples, 1 mL of phosphoric acid (0.5% v/v) solution, and a small steel ball were added to the EP tube. The samples were ground uniformly, then vortexed for 10 min and ultrasonicated for 5 min. 0.1 mL of supernatant was added to a 1.5 mL centrifugal tube after the mixture was centrifuged at 12000 r/min for 10 min at the temperature of 4°C. 0.5 mL methyl tert-butyl ether (containing internal standard) solution was added to the centrifugal tube. The mixture was vortexed for 3 min and ultrasonicated for 5 min. Then, the supernatant was collected after 10 min centrifugation at a speed of 12000 r/min at 4°C. Agilent 7890B gas chromatograph coupled to a 7000D mass spectrometer with a DB-FFAP column (30 m length x 0.25 mm i.d.x 0.25 um film thickness, J&W Scientific, USA) was employed for GC-MS/MS analysis of SCFAs.

**Figure 1.**
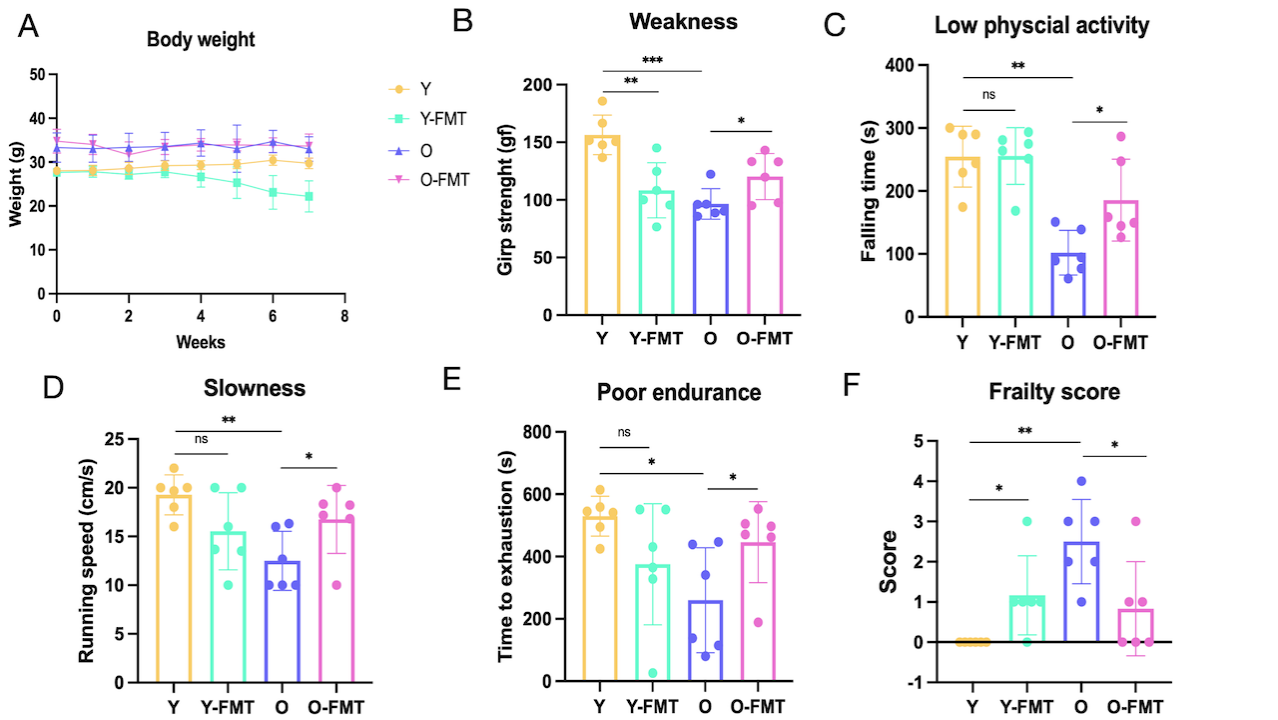
**Frailty score**. (**A**) Weight change. (**B**) Grip strength test. (**C**) Rota-Rod test. (**D-E**) Incremental treadmill assessment of maximum running speed and maximum running time. (**F**) Frailty score. Y group: Young mice (5-6 months) were given PBS, Y-FMT: Young mice (5-6 months) were given fecal microbial transplantation of aged donor microbiota, O: aged mice (22-23 months) were given PBS, O-FMT: aged mice (22-23 months) were given fecal microbial transplantation of young donor microbiota. N=6/group. Data bars represent mean±SD. Dots represent individual data. *p < 0.05, **p < 0.01, ***p < 0.001.

### LC/MS analysis

Liquid chromatography conditions. The LC analysis was performed on a Vanquish UHPLC System (Thermo Fisher Scientific, USA). Chromatography was carried out with an ACQUITY UPLC ® HSS T3 (2.1 × 100 mm, 1.8 μm) (Waters, Milford, MA, USA). The column was maintained at 40 °C. The flow rate and injection volume were set at 0.3 mL/min and 2 μL, respectively. For LC-ESI (+)-MS analysis, the mobile phases consisted of (B1) 0.1% formic acid in acetonitrile (v/v) and (A1) 0.1% formic acid in water (v/v). Separation was conducted under the following gradient: 0~1 min, 8% B1; 1~8 min, 8%~98% B1; 8~10 min, 98% B1; 10~10.1 min, 98%~8% B1; 10.1~12 min, 8% B1. For LC-ESI (-)-MS analysis, the analytes were carried out with (B2) acetonitrile and (A2) ammonium formate (5 mM). Separation was conducted under the following gradient: 0~1 min, 8% B2; 1~8 min, 8%~98% B2; 8~10 min, 98% B2; 10~10.1 min, 98%~8% B2; 10.1~12 min, 8% B2.

Mass spectrum conditions. Mass spectrometric detection of metabolites was performed on Orbitrap Exploris 120 (Thermo Fisher Scientific, USA) with an ESI ion source. Simultaneous MS1 and MS/MS (Full MS-ddMS2 mode, data-dependent MS/MS) acquisition was used. The parameters were as follows: sheath gas pressure, 40 arb; aux gas flow, 10 arb; spray voltage, 3.50 kV and -2.50 kV for ESI (+) and ESI (-), respectively; capillary temperature, 325 °C; MS1 range, m/z 100-1000; MS1 resolving power, 60000 FWHM; number of data dependant scans per cycle, 4; MS/MS resolving power, 15000 FWHM; normalized collision energy, 30%; dynamic exclusion time, automatic.

### Statistical analysis

All the data were displayed as mean ± standard deviation (SD). Shapiro-Wilk normality test was used to check the normal distribution of the data. For two group comparisons, the independent samples t-test was used when the data conformed to a normal distribution, and the non-parametric Mann-Whitney U-test was used when the data did not conform to a normal distribution. Statistical analysis for multiple comparisons was performed by one-way analysis of variance or non-parametric Kruskal-Wallis test. The p-values <0.05 was considered statistically significant. All statistical analyses were conducted with SPSS (ver. 26.0, IBM Corporation, NY, USA) and GraphPad Prism 9.

## RESULTS

### FMT improves frailty in old mice

To assess the effects of FMT on frailty, we exchanged fecal microbiota of young mice (5-6 months) and old mice (22-23 months). Behavioral tests were performed at the end of the experiment. Frailty was assessed by five indicators, including loss of weight, weakness, slowness, poor endurance, and low physical activity level.

Weight: We assessed both initial weight and post-intervention weight, and the weight of the young mice receiving the older mice donor microbiome (Y-FMT group) decreased significantly compared to the young control mice (Y group), while the weight of the aged mice receiving the young donor microbiome (O-FMT group) did not change significantly compared to the older control mice (O group).

Grip strength: When the grip strength was tested at the end of the intervention, the grip strength of the Y-FMT group was significantly decreased compared with that of the Y group (p=0.002), while the grip strength of O-FMT group was greatly improved compared with the that of O group (p=0.036) (Fig. 1B).

Rota-Rod test: The first drop time (low physical activity) in mice was assessed using the Rota-Rod test. There was no significant change in the first drop time of the Y-FMT group compared to the Y group. In contrast, the O-FMT group had a significantly increased first drop time compared to the O group (p=0.025) (Fig. 1C). In conclusion, transplanting fecal microbes of young mice can improve the low physical activity of old mice.

Running speed: The incremental treadmill recorded the maximum running speed of the mice. The maximum running speed of the Y-FMT group was lower than that of the Y group, although the difference was not statistically significant (p=0.121). Compared with the Y group, the running speed of the O group was significantly reduced (p=0.008), however, the speed of the aged mice was greatly improved after fecal microbiota transplantation (p=0.028) (Fig. 1D).

Run time: Mice undergo incremental treadmill tests until exhausted to assess endurance, recording the animal's maximum run time. Compared with the Y group, the endurance of the Y-FMT group was not significantly different (p=0.149), while the maximum running time of the O group was significantly reduced (p= 0.010) (Fig. 1E). The maximum run time of the O-FMT group was improved (p=0.016) (Fig. 1E) compared with the O group.

Frailty score: Frailty scores were based on a score scale consisting of five parameters (5% weight loss, less than 20% for other test results). Compared to the young control group, the Y-FMT group had increased frailty scores (p=0.015) (Fig. 1F), mainly in terms of weight loss and reduced grip strength. Compared to the O group, the frailty of the O-FMT group improved (p=0.041) (Fig. 1F), with grip strength, low physical activity, running speed, and maximum run time being statistically significant. Taken together, these data suggest that an aging gut microbiota is associated with frailty and that a younger gut microbiota improves frailty in aged mice.

Novel Object Recognition Test: A new object recognition test was used to assess memory and learning in mice. Compared with the Y group, the memory and learning ability of the Y-FMT group (p=0.003) and O group (p=0.031) was significantly decreased (Fig. 3S). The memory and learning ability of the O-FMT group was improved (p=0.031) (Fig. 3S) compared with the O group. Consistent with previous findings [18, 22], our results also found that FMT improved cognitive function in aged mice.

### FMT improves muscle mass and systemic inflammation

**Figure 2.**
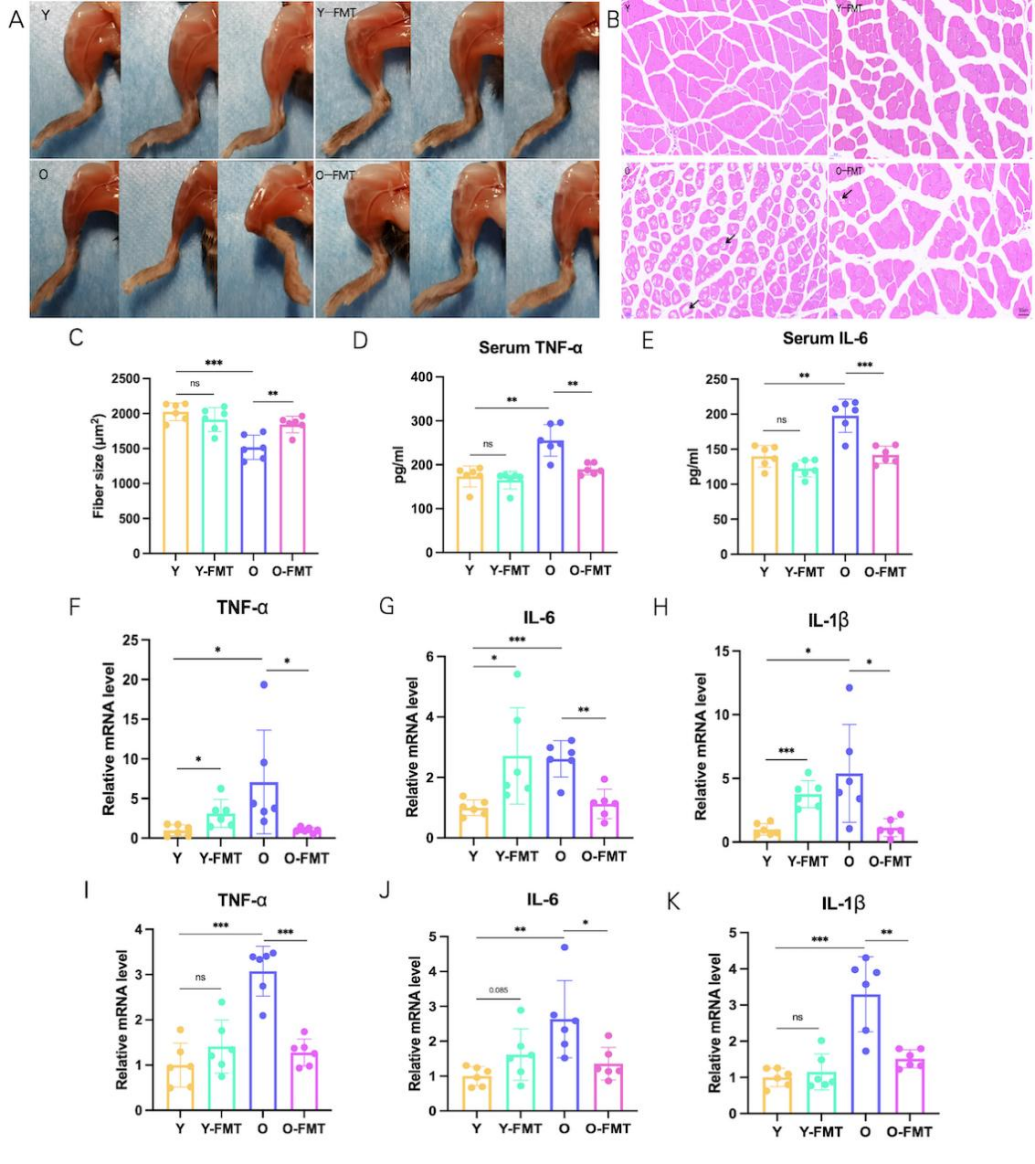
**FMT improves muscle strength and systemic inflammation**. (**A**) Representative photos of the hind limb muscles of each group of mice. (**B**) H&E staining of mice gastrocnemius muscle. The muscles of the old mice atrophied, the muscle septum widened, and the fibrocytes showed focal vacuole-like change (black arrow). (**C**) Gastrocnemius fiber cross-sectional area. (**D-E**) Serum inflammatory factor level. (**F-K**) TNF-α, IL-1β, and IL-6 mRNA levels in the gastrocnemius and colon. N=6/group. *p < 0.05, **p < 0.01, ***p < 0.001.

Inflammation is known to be a driver of frailty, and muscle mass is also strongly associated with frailty. Our results revealed that no significant fibromuscular atrophy and cross-sectional area reduction were observed between the Y group and the Y-FMT group. Compared with the Y group, the muscles of the O group mice atrophied, the muscle septum widened, the myofibre showed focal vacuole-like changes, and the cross-sectional area of the myofibre decreased significantly (p<0.001) (Fig. 2A-C). In contrast, muscle atrophy was ameliorated, and myofibre cross-sectional area was increased (p=0.003) in aged mice that received the young donor microbiota intervention (Fig. 2B-C). The result of Elisa of the key inflammatory cytokines IL-6 (p=0.001) and tumor necrosis factor α (TNF-α) (p=0.001) showed a significant increase in circulating inflammation levels in the O group compared to the Y group, whereas levels of inflammatory factors were significantly reduced after treatment with FMT (TNF-α: p=0.002/IL-6: p<0.001) (Fig. 2D-E). In addition, RT-qPCR results of colon tissues showed that mRNA levels of TNF-α, IL-1β, and IL-6 were elevated in the O group compared with the Y group (TNF-α: p<0.001/IL-1β: p<0.001/ IL-6: p=0.006), and such changes were improved after FMT (TNF-α: p<0.001/IL-1β: p=0.002/ IL-6: p=0.026) (Fig. 2I-K). More importantly, RT-qPCR results of gastrocnemius muscle showed that mRNA levels of TNF-α, IL-1β, and IL-6 were elevated in both the Y-FMT group (TNF-α: p=0.020/IL-1β: p<0.001/ IL-6: p=0.027) and the O group (TNF-α: p=0.047/IL-1β: p=0.019/ IL-6: p<0.001) compared with Y group, suggesting that age-associated intestinal flora may contribute to inflammation in muscle tissue. As expected, FMT can improve the level of muscle tissue inflammation in aged mice (TNF-α: p=0.046/IL-1β: p=0.023/ IL-6: p=0.001) (Fig. 2F-H). Taken together, these data suggested that aging gut microbiota is associated with muscle mass and inflammation levels, all of which can be improved by transplanting young donor microbes.

**Figure 3.**
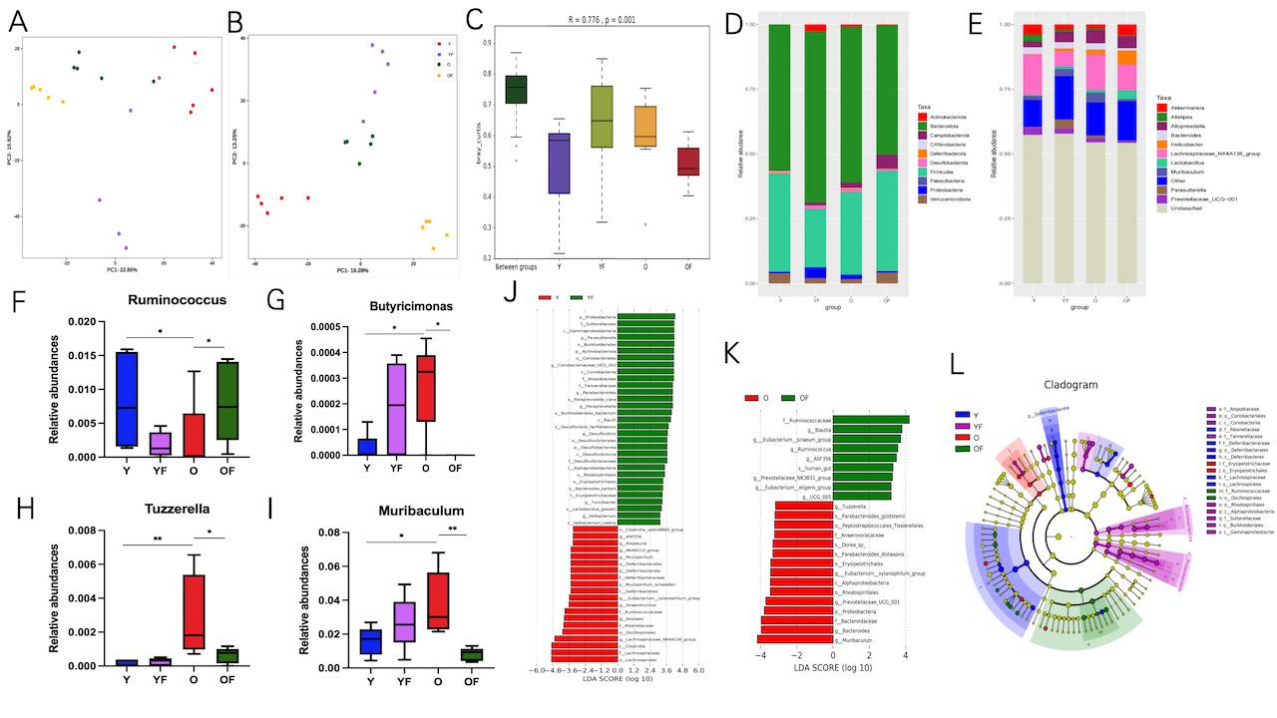
**FMT treatment improves intestinal dysbiosis**. (**A-B**) PCoA plots of β diversity based on the bray-Curtis distance and the Jaccard distance analysis in different groups. (**C**) β diversity based on ANISM analyses. (**D-E**) Top ten of the relative abundance of gut microbiota at the level of phylum and genus. (**F-I**) The relative abundance of four significantly altered bacterial genera. (**J-K**) LEfSe analysis revealed significant differences in the abundance of bacterial taxa between different groups. LDA, Linear discriminant Analysis. (**L**) Graphical phylogenetic analysis of gut microbiota changes. The size of each point represents relative abundance. N= 5/group. *p < 0.05, **p < 0.01, ***p < 0.001.

### FMT reconstructs differences in gut microbiota composition

To explore how FMT administration protects frailty in older mice by modulating microbiome community structure, we performed 16S rRNA sequencing on fecal samples from different groups of mice. Firstly, the abundance and diversity of bacterial species were assessed by α diversity analysis. As shown in Figure S1, we did not observe differences in bacterial diversity or abundance within the groups. Next, we assessed the β-diversity of the groups. Differential clustering of groups of mice was determined in principal coordinate analysis (PCoA) using the Bray-Curtis distance (qualitative measure) and the Jaccard distance (quantitative measure) (Fig. 3A-B). PCoA and ANOSIM analyses (R=0.776, p=0.001, Fig. 3C) revealed a separation of gut microbiota structures between FMT mice and control mice.

Next, we compared the top ten microbiomes in terms of relative abundance at the phylum level and genus level (Fig. 3D-E). At the phylum level, Proteobacteria abundance was increased in the Y-FMT (p=0.009) and O (p=0.075) groups compared to the Y group, while the O-FMT group decreased abundance (p=0.047). In contrast, Verrucomicrobiota was reduced in the Y-FMY and O groups relative to the Y group, although a statistical difference was not reached. However, compared with the O group, the abundance of Verrucomicrobiota was increased with O-FMT (p=0.075). At the genus level, the abundance of Akkermansia and Lachnospiraceae_UCG-001 bacteria in the Y-FMT and O groups was lower than that in the Y group, but the difference was not statistically significant. The abundance of Akkermansia (p=0.075) and Lachnospiraceae_UCG-001 (p=0.047) in the O-FMT group was higher than that in the O group. In contrast, Muribaculum abundances were lower in the Y group and increased in the Y-FMT and O groups (p=0.029), while Muribaculum abundances were lower in O-FMT mice compared to the O group (p=0.009). Then we screened out 4 strains with significant differences between groups (Fig. 3F-I).

LEfSe analysis of microbial characteristics with significant differences among different groups (Fig. 3J-K). Compared with the Y group, the Y-FMT group (LDA score >3.5) had a reduced abundance of Lachnospiraceae_NK4A136_group, Clostridia, Oscillo-spirales, Lachnospirales, and Lachnospiraceae. Among them, Lachnospiraceae_NK4A136_group, Oscillo-spirales, and Lachnospiraceae can promote the production of short-chain fatty acids [23, 24]. In addition, Lachnospiraceae can also affect the host by converting primary bile acids into secondary bile acids and promoting colonization resistance to intestinal pathogens [25]. Compared with the O group, mice in the O-FMT group had an increased abundance of Eubacterium__eligens_group,

Prevotellaceae_NK3B31_group, ASF356, Rumino-coccus, Eubacterium_siraeum_group, Blautia, and Ruminococcaceae. Notably, these bacteria were associated with SCFAs production and reduced inflammation [14, 26-29]. In conclusion, these data suggest that FMT leads to significant changes in the structure of the microbiota, with the characteristic flora of the donor group enriched in recipient mice.

**Figure 4.**
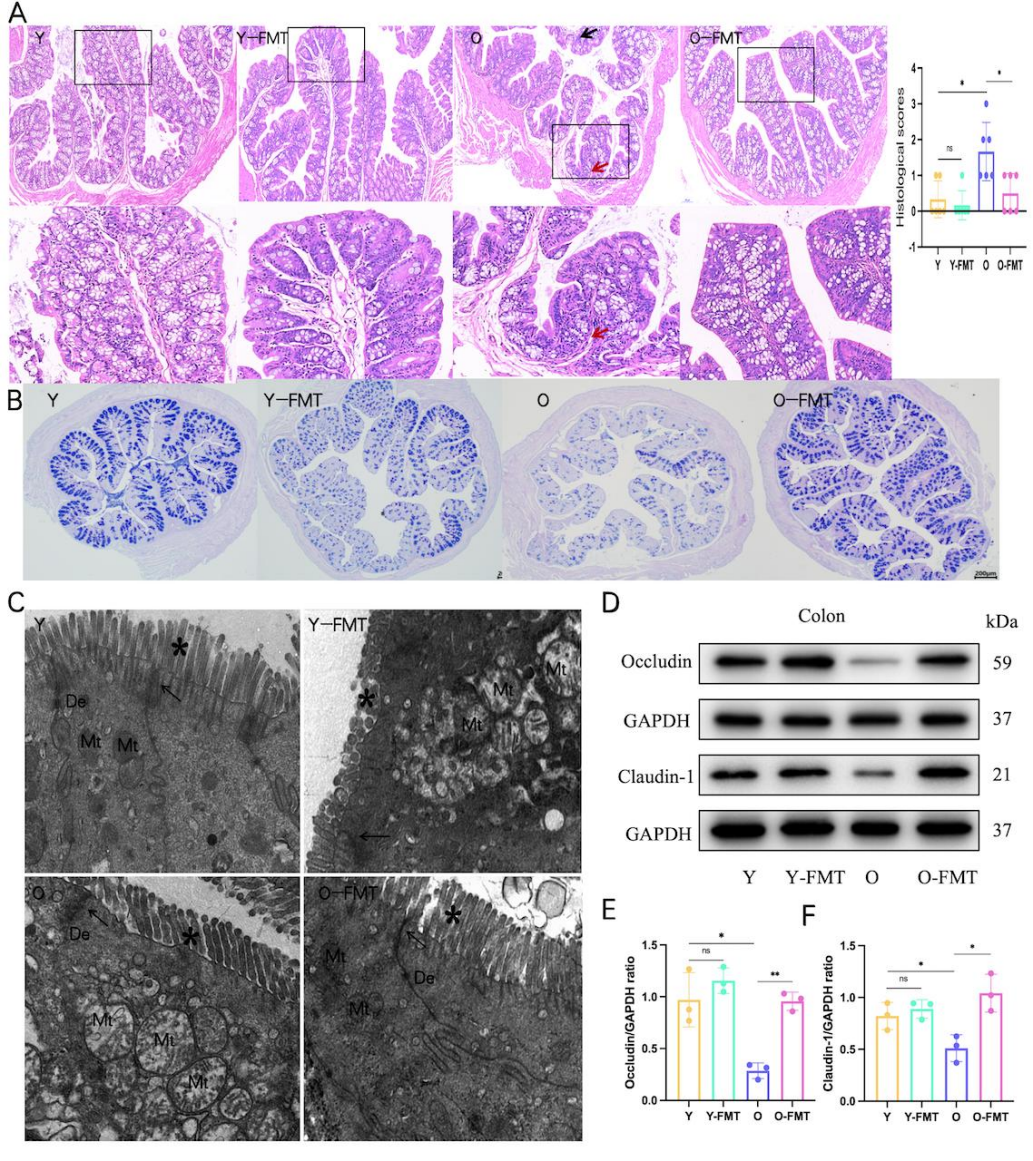
**FMT improves the intestinal barrier damage and inflammation driven by aging gut microbiota**. (**A**) Colon H&E staining and histological scores. The O group showed local crypt atrophy, widening of the spacing, a slight decrease in the number of goblet cells (red arrow), and a small amount of lymphocyte infiltration in the crypt base and mucosal base (black arrow). (**B**) Colon AB-PAS staining. (**C**) Representative TEM of the tight junction structure of the colon epithelium. *: microvilli, arrow: tightly connected structure, De: desmosomes, Mt, mitochondria, Scale bar: 1000 nm. (**D-F**) Density analysis of occludin and claudin-1 blots in the colon. A-B: N=6/group; C:N=3/grorp. *p < 0.05, **p < 0.01, ***p < 0.001.

**Figure 5.**
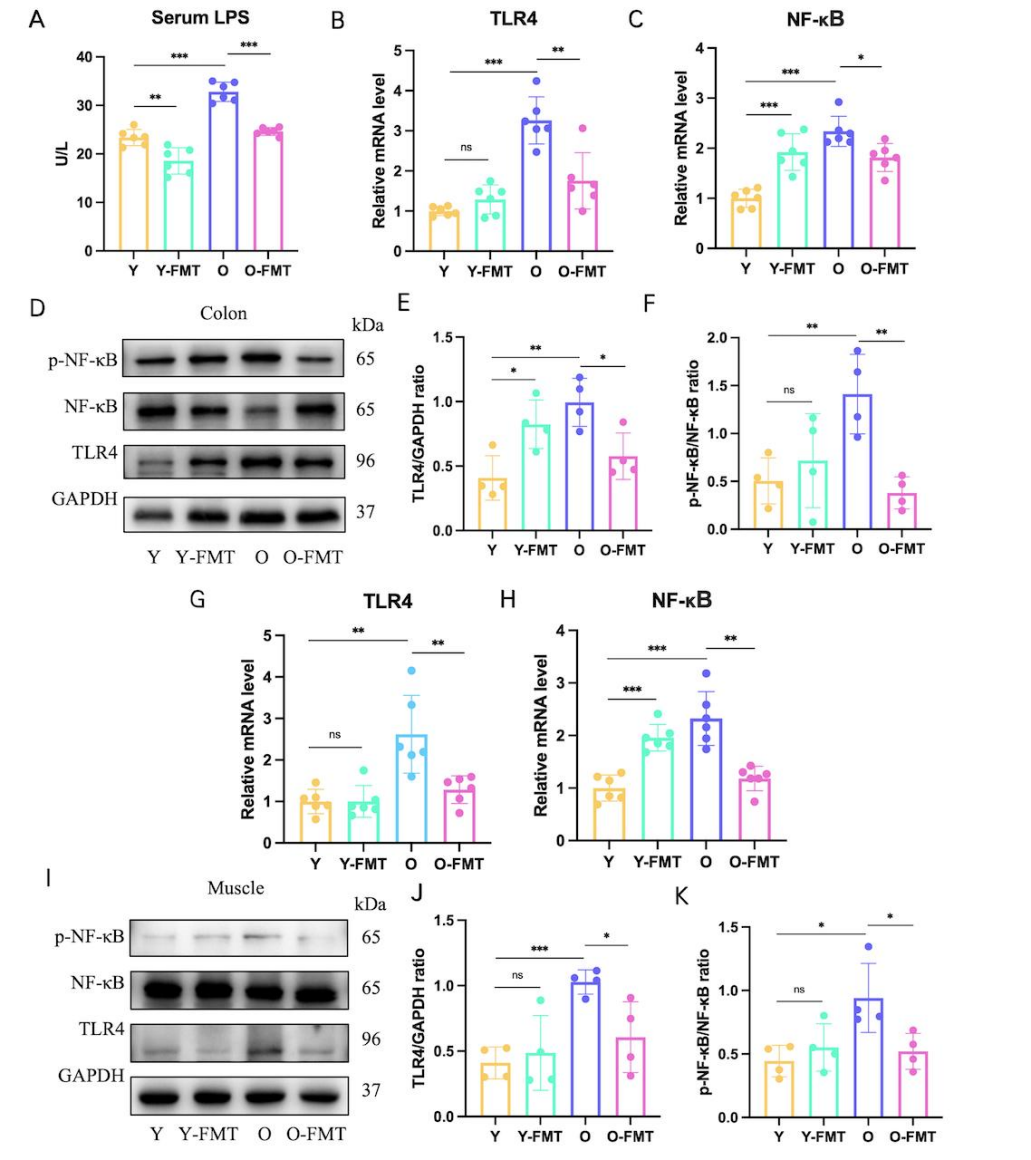
**FMT inhibits intestinal and muscle LPS/TLR4/NF-κB pathways**. (**A**) Serum LPS levels. (**B-C**) mRNA expression of TLR4 and NF-κB in colon tissue. (**D-F**) Protein expression levels of TLR4, p-NF-κB, and NF-κB in colon tissue. (**G-H**) mRNA expression of TLR4 and NF-κB in muscle tissue. (**I-K**) Protein expression levels of TLR4 and p-NF-κB/NF-κB in muscle tissue. For A-C and G-H: N=6/group; For D-F and I-K: N=3-4/group. *p < 0.05, **p < 0.01, ***p < 0.001.

### Healthy FMT improves intestinal barrier damage in frailty mice

Intestinal barrier damage is closely related to frailty. To further explore the role of FMT in the intestinal barrier, we performed H&E, AB-PAS staining, TEM, and western blot detection on the colon to evaluate the changes in intestinal structure and function. Colon H&E staining showed that compared with the Y group, the O group mice showed local crypt atrophy, wider spacing, a slight decrease in the number of goblet cells, and a small amount of lymphocyte infiltration in the crypt base and mucosal base. This situation was improved in the O-FMT group. H&E histological scores were significantly higher in group O (p=0.011) than in Y, while O-FMT significantly reduced scores (p=0.011) (Fig. 4A). Next, AB-PAS staining showed that Y-FMT and O significantly reduced mucus secretion compared to Y, while O-FMT improved colon mucus secretion (Fig. 4B). TEM analysis was performed to detect the structure of the intestinal barrier (Fig. 4C). In the Y group, the colonic microvilli and the compact connective structure were closely connected, and the desmosomes and mitochondria were normal, while in the Y-FMT group, the colonic microvilli were sparse, the compact connective structure was slightly wider, and the mitochondria were swollen. Similarly, the old mice also showed the widening of the focal tight junction structure, the focal shortening and loss of the microvilli structure, the mitochondrial swelling, and the mitochondrial ridge almost disappeared. However, after FMT treatment, the epithelium had regular organized microvilli and intact tight junctions, with intact desmosomes and normal mitochondrial structure. What’s more, blotting results showed that compared with Y group mice, the expression of occludin (p=0.012) and claudin-1 (p=0.043) in colon samples of O group mice decreased significantly, but increased after FMT intervention (occludin, p=0.001; claudin-1, p=0.018) (Fig. 4D-F). Overall, these results suggest that FMT treatment significantly improved intestinal structural and functional impairment in older mice.

### FMT improves frailty by inhibiting the gut-muscle axis LPS/TLR4/NF-κB pathway

We hypothesize that the broken barrier leads to the leakage of pathogenic LPS, which activates the TLR4 signaling pathway in the colon and muscle. To test this hypothesis, we first detected circulating LPS levels, and Elisa’s results showed that LPS in the O group was higher than that in the Y group (p<0. 001), but LPS levels were significantly reduced in the O-FMT group of mice (p<0.001, Fig. 5A). TLR4 and its downstream pathway can recognize elevated LPS, and TLR4 (p=0.004) and p-NF-κB/NF-κB (p=0.009) protein expressions were increased in group O compared with group Y. In the O-FMT group, TLR4 (p=0.018) and p-NF-κB/NF-κB (p=0.004) protein expressions were decreased (Fig. D-F). Again, mRNA levels of TLR4 and NF-κB confirmed this idea (Fig. 5B-C). Similarly, we measured TLR4 activation in muscle tissue, and the results were generally consistent with those in muscle tissue. Compared with group Y, TLR4 (p<0.001) and p-NF-κB/NF-κB (p=0.016) protein expression increased, while TLR4 (p=0.026) and p-NF-κB/NF-κB (p=0.034) protein expression decreased in O-FMT group (Fig. 5I-K). Compared with group Y, mRNA levels of TLR4 (p=0.002) and NF-κB (p<0.001) were also increased in group O and decreased after FMT treatment (TLR4: p=0.008/NF-κB: p=0.001, Fig. 5G-H). All evidence supported FMT administration in the aged to inhibit the LPS-activated TLR4 /NF-κB signaling pathway in colon and muscle tissue.

### FMT alters fecal metabolites

**Figure 6.**
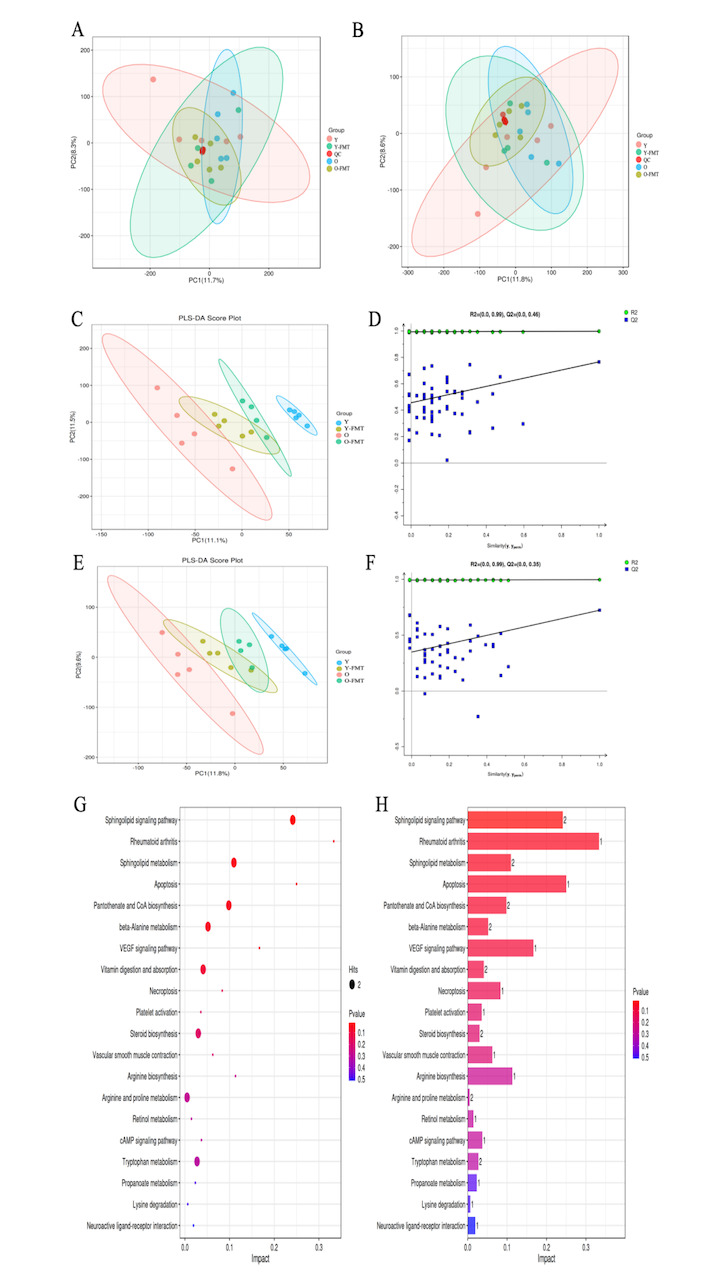
**The analysis results of fecal metabolites**. (**A**) PCA analysis in the POS positive ions mode. (**B**) PCA analysis in the NEG negative ions mode. (**C-D**) PLS-DA analysis in the POS positive ions mode. (**E-F**) PLS-DA analysis in the NEG negative ions mode. (**G-H**) KEGG pathway enrichment analysis. N=4-5/group.

Differences in the overall distribution of the samples in each group were observed by unsupervised principal component analysis (PCA) analysis (Fig. 6A-B). In the POS positive ions mode and NEG negative ions mode, the QC samples were significantly clustered and slightly deviated from the center of the PCA plots, indicating better stability and reproducibility. Partial least squares discriminant analysis (PLS-DA) showed significant differences in metabolites among four groups (Fig. 6C, 6E). The replacement test plots of PLS-DA showed (Figs. 6D,6F) that both R2 and Q2 of the stochastic model gradually decreased with the gradual decrease of replacement retention, indicating that the model was not overfitted and the model was stable. The PLS-DA analysis showed that there were significantly different distribution trends in the Y, Y-FMT, O, and O-FMT groups, indicating that there were significant differences in metabolites among the groups. KEGG pathway enrichment analysis of the differential metabolite was performed using MetaboAnalyst. The results revealed that the differential metabolites were significantly enriched in the following pathways: sphingolipid metabolism pathway (p=0.016), rheumatoid arthritis (p=0.040), and sphingolipid metabolism (p=0.044) (Fig. 6G-H). Based on the analysis of the above results, we speculated that the mechanism by which FMT ameliorates frailty in aged mice may be related to the modulation of sphingolipid metabolic pathways.

### FMT improves short-chain fatty acid levels

Next, we examined the levels of SCFAs in the stool samples (Fig. S2). Acetic acid levels in Y-FMT (p=0.010) and O (p<0.001) groups decreased relative to group Y, while O-FMT increased acetic acid levels, although not reaching statistical significance. Butyric acid levels were elevated in the Y-FMT group (p=0.018) relative to the Y group but were not statistically significant between the O and O-FMT (p=0.084) groups. Propionic acid levels were reduced in the O group (p=0.011) compared to the Y group but were reversed in the O-FMT group (p=0.028) with a statistically significant difference. The level of caproic acid was higher in the Y group and lower in the Y-FMT (p=0.002) group, indicating that the fecal caproic acid level in the old group could be reduced. Although caproic acid levels were lower in the O group (p=0.040) than in the Y group, FMT did not increase caproic acid levels in old mice. SCFAs are the products of intestinal flora digestion and metabolism. They play a local role in the mucosal layer, maintain intestinal function and barrier integrity, increase the protective mucus layer, regulate T lymphocytes, and may enter the systemic circulation to regulate cytokine secretion [30]. Its beneficial effects have been demonstrated in several age-related diseases, including high blood pressure, diabetes, atrial fibrillation, and stroke [31]. Based on these results, we speculate that SCFAs may also be another key target for FMT to ameliorate frailty.

### SCFAs improve frailty

**Figure 7.**
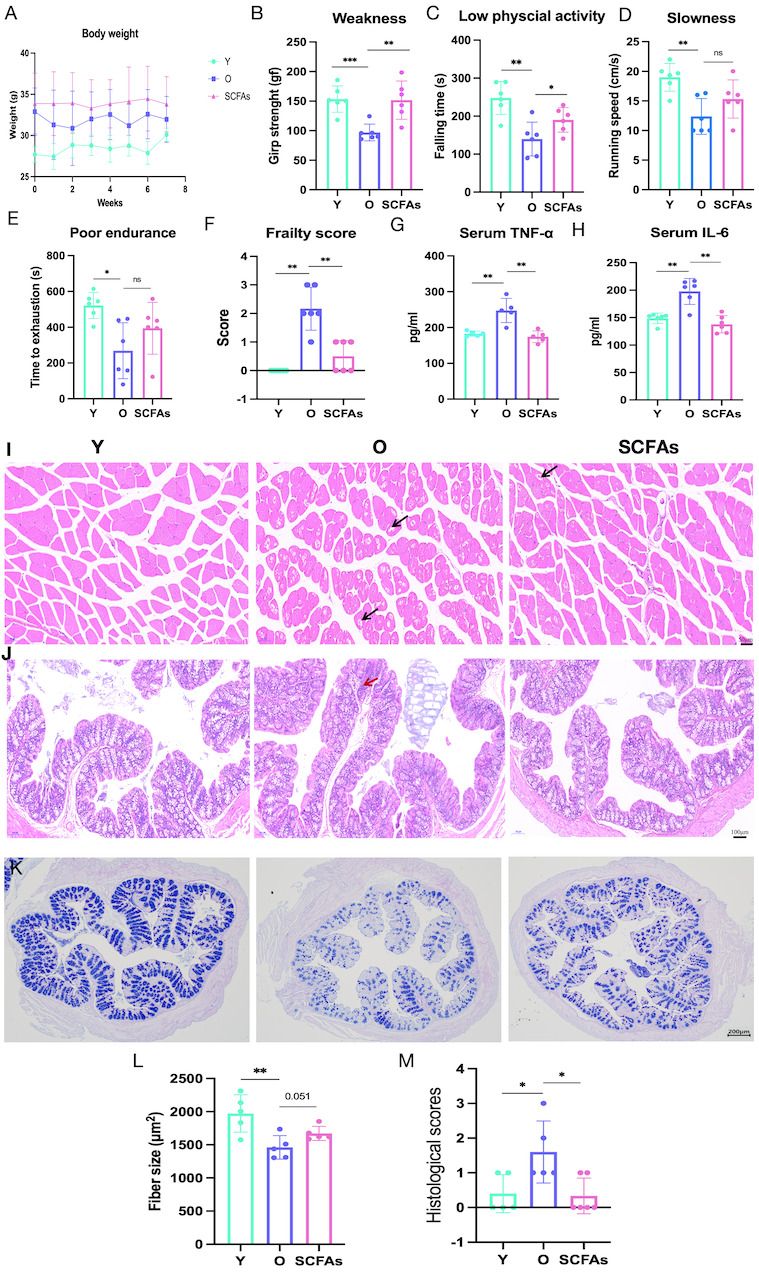
**SCFAs improve frailty**. (**A**) Weight change. (**B**) Grip strength test. (**C**) Rota-Rod test. (**D-E**) Incremental treadmill assessment of maximum running speed and maximum running time. (**F**) Frailty score. (**G-H**) Serum TNF-α and IL-6 levels. (**I**) H&E staining of mice gastrocnemius muscle. (**J-K**) Colon H&E, and AB-PAS staining. (**L**) Gastrocnemius fiber cross-sectional area. (**M**) Colonic histopathological score. N=5-6/group. *p < 0.05, **p < 0.01, ***p < 0.001

To further verify whether the beneficial effect of FMT on frailty in older mice is mediated by improved levels of SCFAs, we gave aged mice drinking water supplemented with SCFAs for 6 weeks and then assessed frailty scores, intestinal barrier, and muscle mass. Results showed that SCFAs improved weight loss, grip strength (p=0.003), first drop time (p=0.034), running speed (p=0.134), maximum running time (p=0.262), and frailty scores (p=0.004) in aged mice, but running speed and maximum running time did not reach statistical significance (Fig. 7A-F). Overall, SCFAs can improve frailty in older mice. In addition, SCFAs reduced the levels of circulating inflammatory factors (TNF-α: p=0.009 and IL-6: p=0.006, Fig. 7G-H), and H&E staining in muscles showed that SCFAs improved muscle atrophy and the cross-sectional area of the myofibre (p=0.051, Fig. 7I, 7L). H&E staining in intestines showed SCFAs improved pathological changes and histological scores in aged mice (p=0.018, Fig. 7J, 7M). Subsequent intestinal AB-PAS staining (Fig. 7K) suggested that SCFAs improved mucus production in older mice. Based on the above results, we found that FMT may also improve frailty by increasing the level of SCFAs.

## DISCUSSION

Although recent studies suggest that the microbiome may influence key pathways critical to the pathophysiology of frailty [4], it remains unknown whether altered gut microbiota composition is a therapeutic target for age-related frailty. In the present study, we used bidirectional fecal bacterial transplantation in young and old mice to assess the effects of gut microbiota and its associated metabolites on frailty and its mechanisms in old mice. We found that fecal bacteria transplanted from old mice into young mice reduced body weight and grip strength, and led to elevated inflammatory factors in young mice, but had no significant effect on intestinal barrier function. Notably, fecal bacteria transplanted from young mice into older mice not only improved frailties and muscle mass in older mice, but also improved intestinal ecological imbalances, intestinal barrier function, and systemic inflammation. Previous studies have shown that circulating LPS is an important component of gut microbiome metabolism and rises in circulation with age. Once in the bloodstream, circulating endotoxins promote systemic inflammation, which may trigger a decline in muscle function, thereby impairing physical function and ultimately reducing the quality of life [32]. This is consistent with our finding that increased circulating LPS in aged mice activates gut and muscle TLR4 receptors, leading to an increase in phosphorylation of its downstream protein NF-KB, which further causes an increase in the levels of inflammatory factors, and ultimately to a decrease in muscle mass (low grip strength). Circulating LPS and inflammatory factor levels were reduced and muscle mass improved in the older group after receiving the young donor microbiota intervention. These findings suggested that FMT may ameliorate frailty in aged mice through the microbiota-gut-muscle axis LPS/TLR4 pathway. In addition, short-chain fatty acids are important and beneficial metabolites of intestinal flora. Previous studies have shown that butyrate improves skeletal muscle atrophy in diabetic nephropathy [33]. In our study, older mice given fecal microbial transplants from young mice showed a significant increase in the abundance of SCFA-producing bacteria and increased levels of short-chain fatty acids. Subsequently, further experiments with SCFAs supplementation showed that it had beneficial effects on frailty and muscle mass in older mice. Our data above supported the viewpoint that alterations in the aging-associated gut microbiota lead to gut metabolite disruption, barrier dysfunction, and systemic inflammation, potentially helping to drive a chronic inflammatory state across systems leading to frailty. Whereas FMT ameliorates frailty in aged mice by improving short-chain fatty acid levels and targeting the gut-muscle axis through the LPS/TLR4 pathway. Therefore, the regulation of short-chain fatty acids metabolism and targeting of the gut-muscle axis by the LPS/TLR4 pathway through alteration of gut microbial composition may be potential targets for the treatment of age-related frailty.

### Effect of FMT on aging gut microbiota

The potential for gut microbiota to influence health is particularly relevant for older people. That's because the microbiome can regulate age-related changes in innate immunity, muscle loss, and cognitive function, all of which are factors in frailty [34]. Studies have shown that with age, the microbiome composition changes composition and function, which leads to reduced gut microbiome biodiversity and a state of microbial dysbiosis, accompanied by the loss of core symbiotic bacterial species and the expansion of opportunistic microbes [35]. Previous studies have shown that Firmicutes and Verrumicrobia are lower in frailty in older adults at the phylum level and that both phyla dominate the gut flora of healthy humans [35]. This is consistent with our results, which showed that at the phylum level, Firmicutes and Verrumicrobia abundance was higher in the Y group, but decreased in the Y-FMT and O groups, while this was reversed in the O-FMT group (Fig. 3D). This suggested that FMT can reverse the abundance of beneficial bacteria in older mice. In addition, based on consistent findings reported by 10 case-control studies and one cohort study, frailty older adults have a lower relative abundance of phylum Firmicutes and genera Ruminococcus [9]. Our results reaffirmed this view, at the genus level, Ruminococcus was more abundant in the Y group, with a decrease in abundance in the O and Y-FMT groups, whereas in the O-FMT group, the abundance of this beneficial genus of bacteria increased. It was found that Ruminococcus possesses anti-inflammatory properties and ameliorates the severity of inflammation in a mouse model of colitis [36]. In addition, Ruminococcus can produce SCFAs (propionate, acetate, and formate), tryptophan, and bile acid metabolites, which contribute to metabolic disturbances in the organism [37]. What is more, Akkermansia and Lachnospiraceae_UCG-001 abundance was also elevated in the O-FMT group. There is growing evidence that Akkermansia exhibits positive systemic effects on host health, mainly improving immune and metabolic functions, while also modulating gut barrier function and intestinal homeostasis [38-40]. Akkermansia also exerts beneficial effects on the organism through the production of acetate and propionate [38, 39]. Lachnospiraceae UCG-001 was found to be positively correlated not only with physical activity and diet [41] but also negatively correlated with the inflammatory factor IL-1β [42]. All of these reasons may be closely related to FMT improving frailty. LEfSe analysis showed that the dominant genera in the O-FMT group include Eubacterium_eligens_group, Prevotellaceae_NK3B31_group, ASF356, Ruminococcus, Eubacterium__siraeum_group, Blautia, and Ruminococcaceae. More interestingly, all these bacteria can produce SCFAs. SCFAs are important fuels for intestinal epithelial cells and are involved in the regulation of differentiation and functions of enteroendocrine cells, influencing gut motility and enhancing gut barrier functions and host metabolism [26].

Identifying the association of the gut microbiome with frailty opens the door to interventions to improve or prevent disease. Ghosh et al. found that a Mediterranean diet intervention alters the gut microbiome in older adults, reducing frailty and improving health [43]. Theou et al. found that inulin and oligofructose reduced frailty in nursing home residents [44.] Although bacteriotherapy has been used to prevent frailty and unhealthy aging, however, the evidence for flora treatment is in its infancy compared to other interventions [45]. Evidence for the role of microorganisms in frailty treatments is still limited, and our study added new evidence that fecal transplantation can enrich beneficial taxonomic flora, reduce the abundance of harmful flora, and ameliorate frailty in aged mice.

### FMT intestinal barrier damage and inflammation driven by aging gut microbiota

In addition, age-related microbial dysbiosis is also associated with intestinal inflammation and damage to the intestinal barrier, which leads to the translocation of microbial products and toxins into the circulation, which is thought to be a contributing factor to inflammatory aging in older people [35]. In our study, colon RT-qPCR and western blot results confirmed that compared with the young control group, intestinal inflammation was increased intestinal barrier protein expression was decreased in the older mice, and FMT could improve intestinal damage caused by intestinal flora imbalance in aged mice. This was confirmed by the results of HE staining and mucus staining of the colon. More importantly, transmission electron microscopy analysis of the tight junction protein suggested that FMT administration may protect the intestinal barrier by restoring the function of the tight junction.

It is becoming increasingly clear that inflammation and damage to the gut barrier caused by dysregulation of the gut flora promote pro-inflammatory microbial products and cytokines to cross the damaged barrier into circulation, resulting in elevated systemic pro-inflammatory cytokines [35]. Pro-inflammatory cytokines induce muscle degeneration via the ubiquitin-proteasome pathway, which is thought to be a potential factor in weakness [46]. In this study, Elisa’s results of pro-inflammatory microbial products LPS and inflammatory factors (TNF-α and IL-6) in serum confirmed that pro-inflammatory factors leaking into the circulation can induce systemic inflammation. In addition, physical function tests, RT-qPCR, and H&E staining of gastrocnemius muscle and fibromuscular cross-sectional area analysis in mice showed the grip strength of aged mice was decreased, the level of inflammatory factors in muscle tissue was increased, and muscle fiber atrophy was observed. However, this damage could be improved by FMT. Thus, our findings suggest that persistent gut barrier damage associated with dysregulation of the gut microbiome may leak microbial metabolites LPS and pro-inflammatory cytokines into the circulation, leading to systemic inflammation and muscle damage. However, FMT treatment improved intestinal barrier damage and suppressed the leakage of inflammatory factors, thereby suppressing systemic inflammation and improving muscle mass and frailty.

### FMT improves frailty by modulating the LPS-TLR4 pathway

To further explore the specific mechanisms by which FMT protects frailty, we focused on how inflammation mediates communication between these two distant organs, as inflammation plays a key role in the gut-muscle axis. Elisa’s analysis confirmed that the serum LPS level of the aged mice increased significantly. Subsequently, increased LPS was recognized by the TLR4 receptor and then stimulated the NF-κB signaling pathway in muscle tissue. As a downstream product of NF-κB, the production of pro-inflammatory cytokines (TNF-α, IL-1β, and IL-6) can be increased by activation of the TLR4 signaling pathway, which was also detected in tissues in the colon in our study. In contrast, FMT treatment significantly reduced LPS levels, thereby inhibiting the TLR4/NF-κB signaling pathway and subsequent inflammatory response. Overall, our study suggested that FMT ameliorates frailty in aged mice possibly by reducing intestinal barrier damage leading to LPS leakage and inhibiting the TLR4/NF-κB pathway and its downstream inflammatory factors.

In addition, KEGG enrichment analysis showed that FMT may ameliorate Frailty through the sphingolipid metabolism pathway. Sphingolipids are complex lipids that form the building blocks of eukaryotic cell membranes regulate cell function and play an important role in the TLR4 pro-inflammatory signaling pathway [47, 48]. Some sphingolipids, such as sphingomyelin, have long been thought to promote TLR4 signaling in macrophages. Sphingosphingoids may affect the homeostasis level of TLR4 in the plasma membrane, or affect the early LPS-induced internalization of TLR4 or the relatively late stage of activation, i.e. the release of IL-6 after its transcription and translation [49]. Olona et al. Found that sphingosphingoid and some ceramide subspecies promote TLR4 signaling by regulating lipid rafts and TLR4 internalization in macrophages during the early pro-inflammatory phase of LPS-activated cells [47]. Moreover, studies have found that LPS increase synergistically affects the sphingolipid signaling pathway by activating TLR4 signaling, and further enhances the expression of inflammatory cytokines in immune cells [50]. Taken together, the sphingolipid metabolism pathway may play a synergistic role in the LPS/TLR4 pathway. In combination with the current findings, we found that LPS/TLR4/NF-κB pathway activation synergistic sphingoid metabolic signaling pathway may be a potential target for FMT improve frailty.

### FMT can regulate SCFA levels

In our study, FMT improved the microbiota structure and composition in older mice. Compared with young mice, the abundance of SCFAs-producing bacteria in old mice decreased, while fecal bacteria transplantation intervention increased the abundance of SCFAs-producing bacteria. Blautia and Prevotellaceae_ NK3B31_group are acetic acid-derived bacteria, while Ruminococcaceae and Eubacterium__siraeum_group are butyric acid-producing bacteria [14, 26]. Ruminococcus, Eubacterium__eligens_group, and ASF356 can also produce the main components of SCFAs [26, 27, 51]. Most of these bacteria with varying relative abundances were successfully transferred to older recipient mice via FMT. In addition, GC-MS/MS analysis showed increased levels of short-chain fatty acids in older mice treated with FMT, although only the difference in propionic acid levels reached statistical significance.

Short-chain fatty acids produced by fermentation of the gut microbiota are known to have antioxidant properties, immunomodulatory functions, and regulation of energy metabolism [30]. Walsh et al. found that butyrate can reduce muscle atrophy in older mice and prevent fat accumulation in muscles [52]. Lv et al. found that the human gut microbiome affects skeletal muscle mass in healthy postmenopausal women through the synthesis of short-chain fatty acid butyrate by gut microbes [53]. Therefore, we speculate that the beneficial effect of fecal bacteria transplantation on the improvement of frailty in old mice may also be through short-chain fatty acid homeostasis. We then referred to another study [14], which further validated the effects of SCFAs by continuously supplementing older mice with drinking water containing a mixture of the three main components of SCFAs. Short-chain fatty acids intake altered frailty in older mice while improving gut barrier function and inflammation.

By investigating the potential mechanism of FMT's protective effect on Frailty, this study partially explains the role of the microbiota-gut-muscle axis in frailty. Although a growing body of evidence confirms that gut microbiota imbalance and barrier damage are associated with frailty in old age, the specific pathways and mechanisms of interaction between gut and frailty remain unclear. Based on our current results, we speculate that LPS, a metabolite derived from gut microbiota metabolism, can leak pro-inflammatory factors into the circulation through activation of TLR4/NF-κB signaling pathway-induced intestinal inflammation and subsequent sustained and relatively severe intestinal barrier disruption, which may lead to chronic inflammation throughout the body and ultimately to decreased bodily function. By regulating intestinal homeostasis imbalance, increasing short-chain fat levels, improving intestinal barrier damage, and inhibiting LPS leakage, FMT alleviated frailty in aged mice. Based on our findings, whether people with unbalanced gut homeostasis are more prone to frailty should be explored in future studies. Our results provide new evidence for the therapeutic role of microorganisms in frailty, and future microbial therapy for the older population needs to be further explored for clinical conversion.

### Conclusion

Our study is the first to reveal the protective effect of FMT treatment on frailty in older mice. Further mechanistic studies suggest that FMT administration reverses gut microbial dysbiosis and improves frailty in older mice, in which inhibition of inflammation mediated by the LPS-TLR4 signaling pathway and increased SCFA levels may play an important role. Therefore, regulating SCFAs metabolism by altering gut microbial composition and targeting the gut-muscle axis with LPS/TLR4 pathways may be potential strategies to treat frailty in older adults.

## Supplementary Materials

The Supplementary data can be found online at: www.aginganddisease.org/EN/10.14336/AD.2024.0321.



## Data Availability

All data generated or analyzed during this study are available from the corresponding author upon reasonable request.
