# Supplementary material for: Fecal Microbiota Transplantation Attenuates Frailty via Gut-Muscle Axis in Old Mice
Source: Aging Dis. 2024 Mar 21;16(2):1180–98. doi: 10.14336/AD.2024.0321 (PMC11964440; doi:10.14336/AD.2024.0321)
Supplement: Supplementary file 1 [file AD-16-2-1180-s.pdf]

SUPPLEMENTARY DATA

Fecal Microbiota Transplantation Attenuates Frailty *via* Gut-Muscle Axis in Old Mice

Mengpei Zhu, Yumei Huang, Ziwen Wang, Ze Jin, Jiali Cao, Qiangqiang Zhong, Zhifan Xiong

© 2024. Zhu M et al. Published online at http://www.aginganddisease.org/EN/10.14336/AD.2024.0321

SUPPLEMENTARY DATA

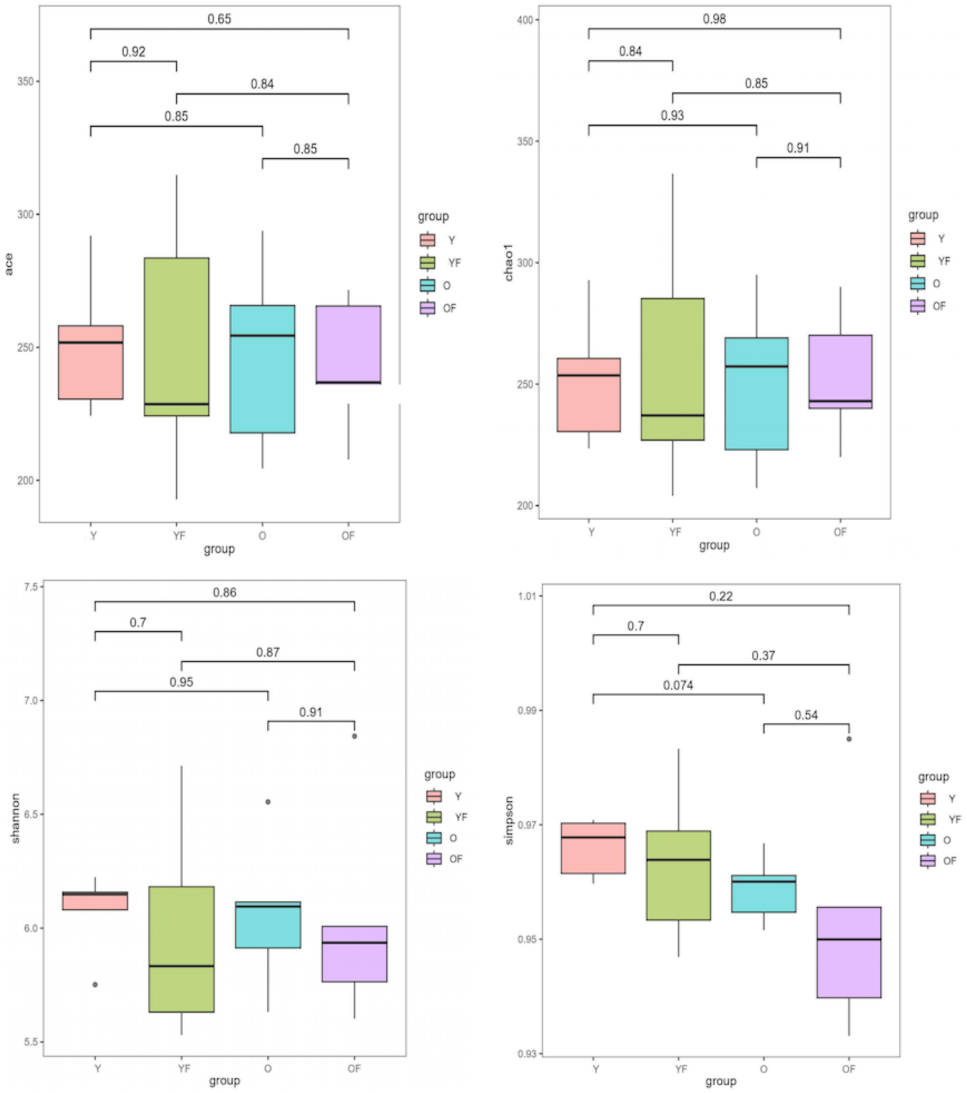

Supplementary Figure 1. Analysis of α diversity of gut microbiota by Ace, chao1, Shannon, and Simpson analysis. N=5/group.

© 2024. Zhu M et al. Published online at http://www.aginganddisease.org/EN/10.14336/AD.2024.0321

SUPPLEMENTARY DATA

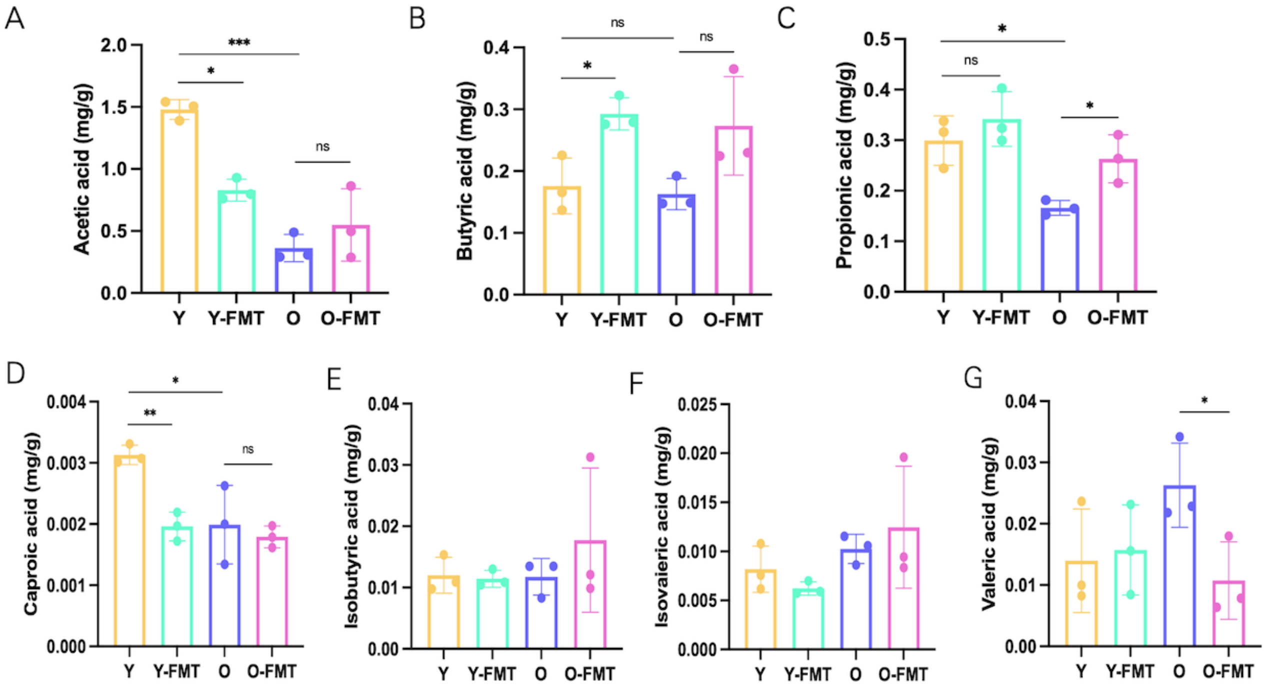

Supplementary Figure 2. FMT improves SCFA levels. (A) Acetic acid level. (B) Butyric acid levels. (C) Propionic acid levels. (D) Caproic acid levels of four groups. (E) Isobutyric acid level of four groups. (F) Isovaleric acid levels of four groups. (G) Valeric acid levels of four groups. N=3/group. *p < 0.05, **p < 0.01, ***p < 0.001.

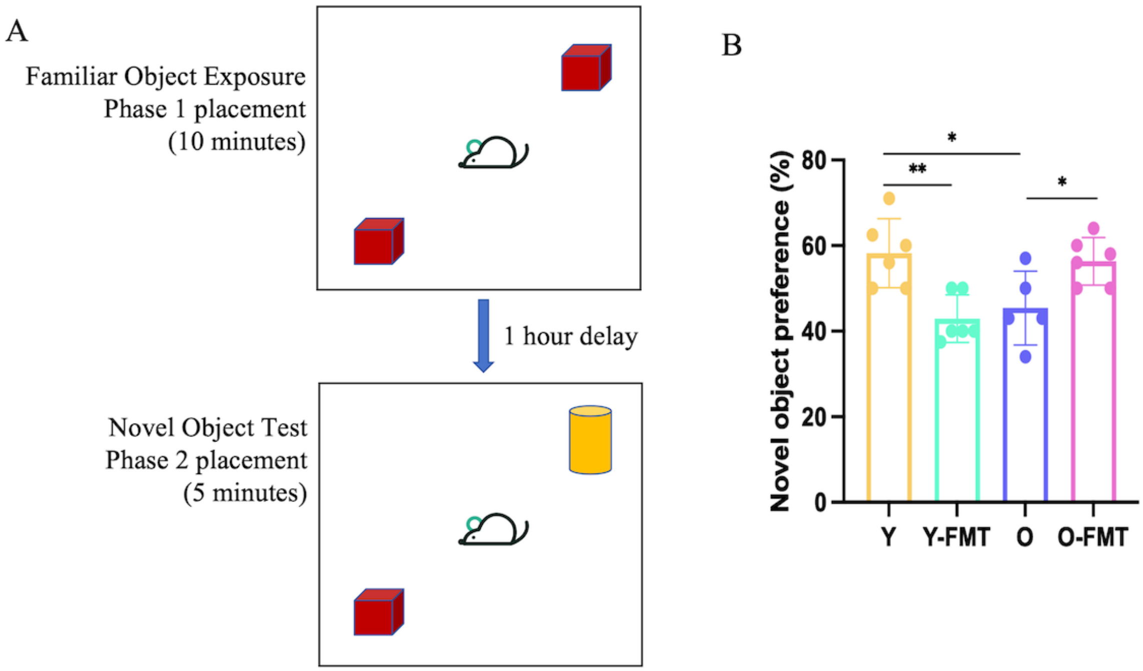

Supplementary Figure 3. Novel Object Test. (A) Schematic diagram of the Novel Object Test. (B) Results of the Novel Object Test. N=5-6/group. *p < 0.05, **p < 0.01, ***p < 0.001.

© 2024. Zhu M et al. Published online at http://www.aginganddisease.org/EN/10.14336/AD.2024.0321
